# A Systematic Review of Varying Definitions and the Clinical Significance of Fredet’s Fascia in the Era of Complete Mesocolic Excision

**DOI:** 10.3390/jcm12196233

**Published:** 2023-09-27

**Authors:** Gioia Brachini, Bruno Cirillo, Matteo Matteucci, Roberto Cirocchi, Giovanni Domenico Tebala, Davide Cavaliere, Lorenza Giacobbi, Veronica Papa, Leonardo Solaini, Stefano Avenia, Vito D’Andrea, Justin Davies, Piergiorgio Fedeli, Elena De Santis

**Affiliations:** 1 Department of Surgery, Sapienza University of Rome, 00161 Rome, Italy; gioia.brachini@uniroma1.it (G.B.); vito.dandrea@uniroma1.it (V.D.); elena.desantis@uniroma1.it (E.D.S.); 2 Department of Medicine and Surgery, University of Milan, 20122 Milan, Italy; matteo.matteucci@unimi.it; 3 Department of Medicine and Surgery, University of Perugia, 06132 Perugia, Italy; roberto.cirocchi@unipg.it (R.C.); lorenza.giacobbi@studenti.unipg.it (L.G.); stefano.avenia@gmail.com (S.A.); 4 Digestive and Emergency Surgery, AOSP of Terni, 05100 Terni, Italy; g.tebala@aospterni.it; 5 General Surgical Department, Ospedale Degli Infermi Faenza, 48018 Faenza, Italy; cavalied@gmail.com; 6 Department of Motor Sciences and Wellness, University of Naples “Parthenope”, 80132 Napoli, Italy; veronica.papa@uniparthenope.it; 7 General and Oncologic Surgery, Morgagni-Pierantoni Hospital, Ausl Romagna, 47121 Forlì, Italy; leonardo.solaini2@unibo.it; 8 Cambridge Colorectal Unit, Addenbrooke’s Hospital, Cambridge University, Hospitals NHS Foundation Trust, Cambridge CB2 0QQ, UK; justindavies2000@yahoo.com; 9 University of Cambridge, Cambridge CB2 0QQ, UK; 10 School of Law, Legal Medicine, University of Camerino, 62032 Camerino, Italy; piergiorgio.fedeli@unicam.it

**Keywords:** right colon, hemicolectomy, CME, colon adenocarcinoma, laparoscopy

## Abstract

Background: Fredet’s fascia represents a crucial landmark for vascular surgical anatomy, especially in minimally invasive complete mesocolic excision (CME) for right-sided colon adenocarcinoma. Fredet’s fascia allows access to the gastrocolic trunk of Henle (GCTH), the most critical step in both open and minimally invasive right-sided CME techniques. Despite this, a recent workshop of expert surgeons on the standardization of the laparoscopic right hemicolectomy with CME did not recognize or include the term of Fredet’s fascia or area. Hence, we undertook a systematic review of articles that include the terms “Fredet’s fascia or area”, or synonyms thereof, with special emphasis on the types of articles published, the nationality, and the relevance of this area to surgical treatments. Methods: We conducted a systematic review up to 15 July 2022 on PubMed, WOS, SCOPUS, and Google Scholar. Results: The results of the study revealed that the term “Fredet’s fascia” is poorly used in the English language medical literature. In addition, the study found controversial and conflicting data among authors regarding the definition of “Fredet’s fascia” and its topographical limits. Conclusions: Knowledge of Fredet’s fascia’s surgical relevance is essential for colorectal surgeons to avoid accidental injuries to the superior mesenteric vascular pedicle during minimally invasive right hemicolectomies with CME. In order to avoid confusion and clarify this fascia for future use, we suggest moving beyond the use of the eponymous term by using a “descriptive term” instead, based on the fascia’s anatomic structure. Fredet’s fascia could, therefore, be more appropriately renamed “sub-mesocolic pre-duodenopancreatic fascia”.

## 1. Introduction

Colorectal cancer represents the third most common cause of cancer death, with 1.8 million new cases in 2018 worldwide [1].

To date, optimal surgery remains a milestone for successful oncological outcomes.

Recent advances in the treatment of colon cancer identify complete mesocolic excision (CME) as a potential determinant of survival following oncological resection. As with total mesorectal excision (TME) for rectal cancer, CME, first proposed by Hohenberger in 2009 [2], is based on sharp dissection following embryological anatomical planes leading to a surgical specimen with intact coverage, not only of the tumor but also of lymphatics and vessels.

CME is combined, indeed, with central ligation of the tumor-supplying arteries and draining veins, referred to as central vascular ligation (CVL), which allows a more radical lymphadenectomy (D3-L) [3].

More recently, CME has been further optimized through the use of minimally invasive techniques such as laparoscopy and robotics [4], which may facilitate dissection and anatomic precision.

However, the actual mesocolic anatomy and its dissection planes are a relatively recent discovery.

Quite surprisingly, for over a century the anatomical description of the mesocolon widely accepted in teaching and all related sciences originated from the observations made by the British surgeon Sir Frederich Trèves in 1885 [5]. According to Trèves, the ascending and descending colon are retroperitoneal viscera due to the regression of their respective mesocolon; conversely, the transverse and sigmoid mesocolon do not undergo involution and persist in adulthood. In line with this classical description, the mesocolon has thus been described for decades as a fragmented and discontinuous structure.

In 2012, this traditional and well-established concept was refuted by Culligan [6], who documented that the right and left mesocola persist into adulthood as distinct anatomical entities, because during fetal development there is a flattening of the retroperitoneum, rather than them regressing. According to this new anatomical paradigm, therefore, the mesentery, the mesocolon, and the mesorectum are actually continuous structures along their entire length, from duodenal–digiunal flexure to pelvic floor, both in fetal and adult age.

Interestingly, these findings confirm those of Austrian anatomist Carl Toldt, who, in 1879 [7], first proposed that the right and left mesocola persist in adulthood and remain separated from the underlying retroperitoneum by a connective dissection plane, later called by Goligher the “fusion fascia of Toldt”. Currently, it is well known that the Toldt fascia lies immediately posterior to the right and left mesocola, where it adheres to the parietal peritoneum of the retroperitoneum to form a cleavage plane.

Most importantly, this new paradigm represents the rationale for surgeons to conduct colonic mobilization as an intact package along the avascular mesocolic planes. In keeping with this, several studies have shown that Toldt’s fascia is the natural embryonic dissection plane for the conduction of complete mesocolic excision (CME) in colon cancer surgery [8].

However, while CME with D3 lymphadenectomy (D3-L) is widely performed for left colon cancer, CME in right colon cancer turns out to be more challenging.

On the right, in fact, the reflection of the lower peritoneal sheet of the transverse mesocolon on the duodenopancreatic peritoneum forms a distinct but not widely known embryological fascial plane: the Fredet fusion fascia [9], which some authors still erroneously identify as Toldt’s fascia.

**Figure 1:**
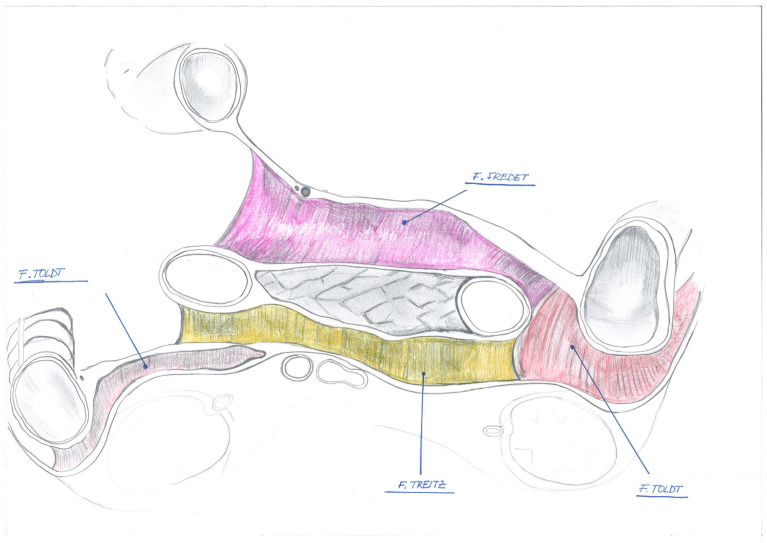
Fredet’s fascia is located in front of the duodenum and pancreas. Embriological fascia is defines as follows: Toldt’s fascia: plane of adhesion between peritoneum of the ascending mesocolon and retroperitoneum; Fredet’s fascia: plane between the visceral peritoneum of the hepatic flexure and pancreas and duodenum; Treitz facia: plane between visceral peritoneum of duodenum and pancreas and retroperitoneum.

**Figure 2:**
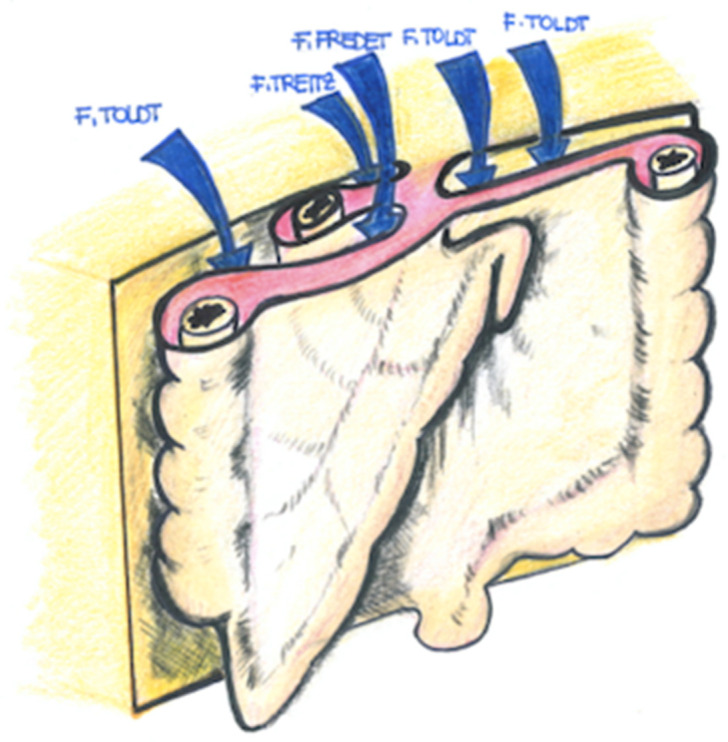
Differences between Toldt’s fascia, Fredet’s fascia, and Treitz fascia.

**Figure 3:**
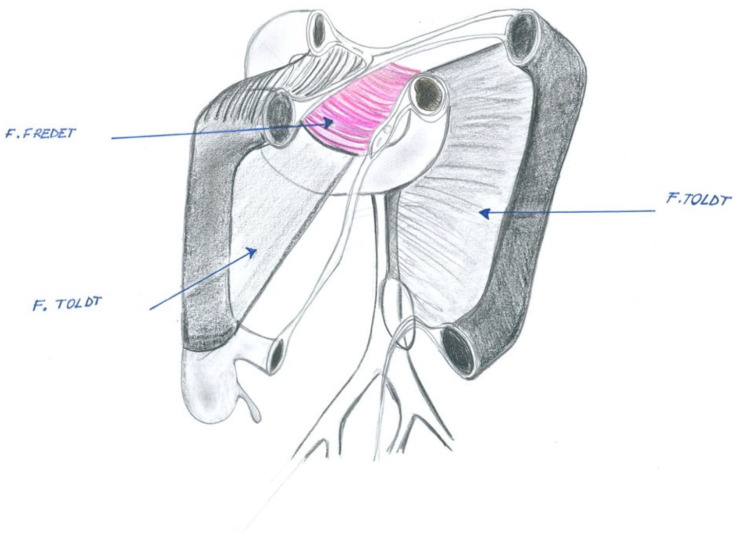
Differences between Toldt’s fascia, Fredet’s fascia, and Treitz fascia.

This embryological plane is named after the French surgeon Pierre Fredet (1870–1946), best known for the Fredet–Ramstedt’s surgical technique for the treatment of newborns with hypertrophic pyloric stenosis. In fact, Fredet also performed original anatomical and embryological studies on renal capsule formation and pre-pancreatic and mesocolic fascia. The French surgeon clearly differentiated this fascia from that of Toldt (Figure 1, Figure 2 and Figure 3).

The real relevance of this fascia, however, derives from its arrangement. In fact, an extended dissection over the descending portion (2nd part) of the duodenum and pancreas head with exposure of the upper mesenteric vessels is necessary for the mobilization of the ascending colon in the context of right-sided CME.

**Figure 4:**
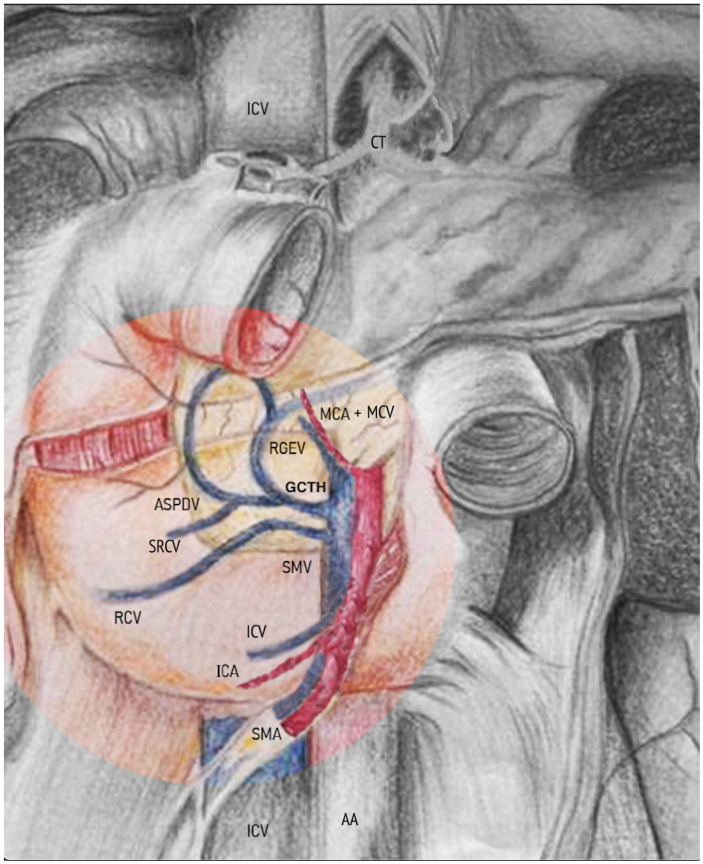
Frontal view of the posterior abdominal wall overlaid by the parietal peritoneum which forms the roots of the transverse mesocolon and small bowel mesentery. Transverse colon and its free mesentery are cut away. The drawing illustrates the anatomical relationships between the “sub-mesocolic pre-duodenal pancreatic fascia” or Fredet’s fascia with the vessels and retroperitoneal organs. The anatomical boundaries/limits of Fredet’s fascia (cranial, caudal, left, right) as well as the gastrocolic venous trunk of Henle (GCTH) described in the text are clearly discernible in the figure. AA abdominal aorta, ASPDV anterior superior pancreatic-duodenal vein, ICV ileocolic vein, ICA ileocolic artery, MCA middle colic artery, MCV middle colic vein, RCV right colic vein, RGEV right gastroepiploic vein, SMA superior mesenteric artery, SMV superior mesenteric vein, SRCV superior right colic vein, GCTH gastrocolic venous trunk of Henle.

Crucially, only the dissection of the Fredet’s fascia allows for the exposure of the submesocolic anterior aspect of the duodenum and pancreas and to enter the surgical area of the gastrocolic venous trunk of Henle (GCTH). The latter is located at the head of the pancreas where it drains into the superior mesenteric vein (SMV). The GCTH represents the most critical point of dissection in both open and minimally invasive right-sided CME techniques. The SMV is located at the medial limit of Fredet’s fascia and represents an important surgical landmark for CME (Figure 4).

Despite its topographical relevance, a recent workshop of expert surgeons on the standardization of laparoscopic right hemicolectomy with CME did not recognize or include the term of Fredet’s fascia or area [10]. The detailed anatomic description of “Fredet’s fascia”, in fact, still needs to be correctly recognized. Moreover, in the medical literature, many authors currently identify the Fredet’s fascia with other eponyms or synonyms.

The aim of this study is, therefore, firstly to clarify the anatomic description of Fredet’s fascia and its relevance in colon cancer surgery, and, secondly, to evaluate the use of the term “Fredet’s fascia” in the current international scientific literature.

## 2. Materials and Methods

We conducted a systematic review up to 15 July 2022 on PubMed, WOS, SCOPUS, and Google Scholar without any language restriction. Our comprehensive searches were performed using the following strategies:

- “Fredet” on PubMed, WOS, and SCOPUS
- “Fredet’s” and “area” or fascia”, and pancreatectomy” on PubMed, WOS, SCOPUS, Google, and Google Scholar
- “Fredet’s” and “area” or fascia”, and “gastrectomy” on PubMed, WOS, SCOPUS, Google, and Google Scholar
- “Fredet’s” and “area” or fascia”, and “colectomy” on PubMed, WOS, SCOPUS, Google, and Google Scholar
- “Preduodenopancreatic” and “fascia” on PubMed, WOS, SCOPUS, Google, and Google Scholar

The search was also performed on additional texts identified through the references of the original articles and relevant grey literature through Google Books (https://books.google.com, accessed on 3 July 2022)

According to the inclusion criteria, we considered only manuscripts in which the term “Fredet” was mentioned in the article/book.

When a database yielded hundreds or thousands of search results, we first read the titles to identify possible records to explore further. Such titles were considered until a point of saturation was reached and the searches yielded results that would clearly not meet the inclusion criteria. Typically, such saturation was reached after reviewing the first 200 search results, as they are often sorted by relevance. We then read the related abstracts of the search results with titles that we thought may lead to publications that met the inclusion criteria; if the abstracts implied that the publication might meet the criteria for inclusion, we then read the entire publication, as to determine whether or not it met the criteria for inclusion.

Our primary outcome of interest was the definition of Fredet’s fascia or area. After extracting the Fredet-related definition(s) from each article, we grouped the definitions into categories using an emergent coding approach and tabulated the frequencies and percentages of each. We did not use advanced statistical synthesis techniques because the usefulness of this review depends on its capacity for describing the variety of anatomical definitions in use rather than generating very precise pooled prevalence and heterogeneity estimates.

Secondary outcomes included the nation where the study was performed, publication source and type (book chapter or journal article; narrative review; observational study; case report; technical note, etc.) and publication topic, (anatomy; surgery: right robotic/laparoscopic colectomy, duodenopancreatectomy, laparoscopic gastrectomy, distal pancreatectomy, laparoscopic intestinal derotation, laparoscopic sigmoidectomy, transverse colectomy, and transplantation).

The protocol for this systematic review was submitted and accepted by PROSPERO: CRD42019122985 (http://www.crd.york.ac.uk/prospero accessed on 3 July 2022).

## 3. Results

**Figure 5:**
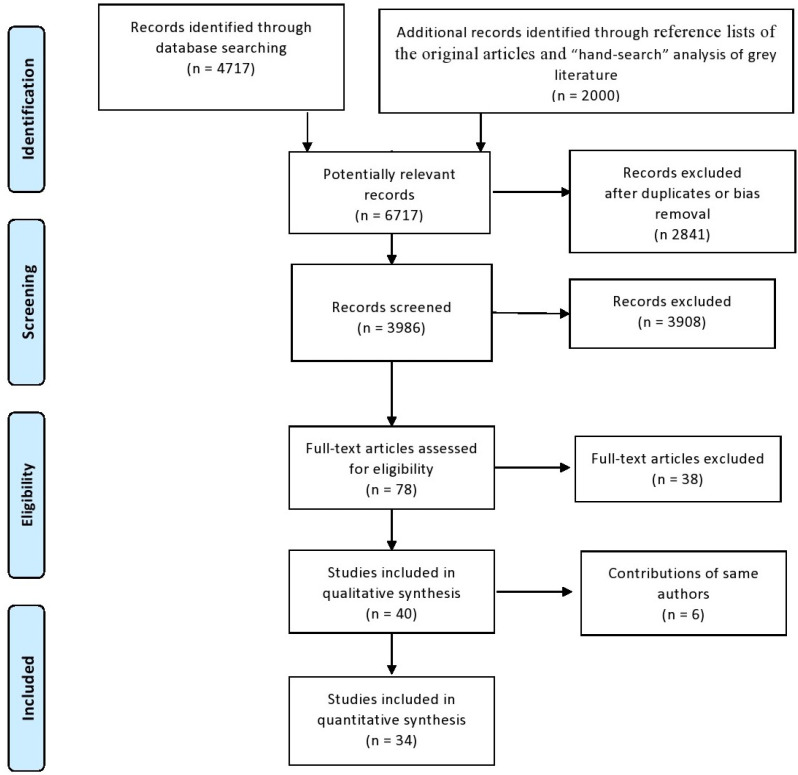
PRISMA flow diagram of study search.

The PRISMA flow diagram shows the results of our systematic search. Ultimately, our search yielded 45 publications [9,11,12,13,14,15,16,17,18,19,20,21,22,23,24,25,26,27,28,29,30,31,32,33,34,35,36,37,38,39,40,41,42,43,44,45,46,47,48,49,50,51,52,53,54] that met the criteria for inclusion (Figure 5), of which 12 were book chapters (26.7%) and 33 were studies (73.3%). There were ten narrative reviews (22.2%), seventeen observational studies (37.8%), three case reports (6.7%), and three technical notes (6.7%) (Table 1). The majority of the publications (*n* = 26) originated from Italy which amounted to 57.8% of the contributions in the field, with 42.2% of the remaining studies originating from 12 other countries: China (*n* = 3), Germany (*n* = 2), Spain (*n* = 2), Argentina (*n* = 2), Greece (*n* = 2), USA (*n* = 2), France (*n* = 1), Romania (*n* = 1), Serbia (*n* = 1), Turkey (*n* = 1), U.K. (*n* = 1), and Japan (*n* = 1).

Among the included publications, the studies from Siani et al. appear five times, the studies from Pugliese, D’Annibale, and Garcia-Granero appear twice.

The term “Fredet’s fascia” was only used by 29 out of 45 (64.4%) authors: different synonyms were reported in the other studies. Investigators used the terms “Fredet’s fascia” and “Fredet pre-duodenal pancreatic fascia” as synonyms in the minority of the studies (11/45, 24.4%). Surgeons claimed that the “*Fredet’s fascia*” definition was equivalent to the “*sub-mesocolic pre-duodenopancreatic fascia*” or “*Fredet area*”; such an area has a precise topographic localization in front of the duodenum and the head of the pancreas, and behind the root of the transverse mesocolon, to the right side of the superior mesenteric vein (SMV). Six studies reported more than one synonym (6/45, 13.3%). Some articles used the terms “*Fredet right transverse mesocolon fascia*” (2/45, 4.4%) and “*Fredet sub-mesocolic duodenal-pancreatic fascia*” (1/45, 2.2%) (Table 2).

The “Fredet’s fascia” was mostly defined as “the area below the right transverse mesocolon as a result of the joining of the inferior layer of the root of the transverse mesocolon to the visceral peritoneum layered onto the duodenum and the pancreas” (12/15, 80%), with few studies (3/15, 20%) reporting that it is the area “below the root of the mesocolon, the fusion of the inferior lining of the transverse mesocolon (right and left) to the ventral surface of the pancreas (body and tail)” (Table 3).

In the articles reporting the term “*Fredet’s fascia*” or one of its synonyms, the vast majority refer to laparoscopic/robotic right colectomy; only a few articles mention other surgical techniques such as pancreaticoduodenectomy, gastrectomy, or distal pancreatectomy (Table 4).

The relevance of Fredet’s fascia in the main surgical techniques used by the authors (such as robotic/laparoscopic right colectomy, duodenal-pancreatectomy, and laparoscopic gastrectomy) is primarily that its sharp dissection in the avascular plane allows the best exposure of the gastrocolic trunk of Henle (GCTH) and of the anterior surface of the SMV. This latter anatomical landmark represents the medial limit of this area (Table 5).

Consequently, “Fredet’s fascia” (“sub-mesocolic pre-duodenopancreatic fascia”) is an important anatomical landmark for the vascular surgical anatomy of the right colic vein, the right gastroepiploic vein, the gastrocolic Henle’s trunk, and the SMV—the most important anatomical landmark in CME for right colon cancer (Table 6).

## 4. Discussion

The latest advancements in surgical oncology for colorectal adenocarcinoma emphasize the crucial role of a radical approach performed along the embryonic developmental planes [8]. Hence the importance of detailed knowledge of these structures and their correct and universal definition. Focusing on the Fredet fascia, this literature review provides convincing evidence of the lack of consensus on the topographical limits and terminology of this embryological plane.

Regarding Fredet’s fascia’s topographical limits, some considerations are necessary. The root of the transverse mesocolon divides the abdominal cavity into two main compartments, the supra and inframesocolic, which are then divided into subspaces. This root is oblique from right to left and from inferior to superior. It crosses the lower third of the right kidney, the descending portion (second part) of the duodenum, and the head of the pancreas; then, it passes over the duodenojejunal flexure and runs along the inferior border of the body and tail of the pancreas to finish at the upper third of the left kidney.

Crucially, the inframesocolic compartment is further divided by the root of the small bowel mesentery, in two unequal spaces: the smaller right inframesocolic space and the larger left submesocolic space. The root of the mesentery starts crossing the duodenum mostly at the level of the union of the horizontal portion (third part) with the ascending portion (fourth part) of the duodenum and continues running obliquely to the ileocaecal valve.

In the right inframesocolic space, the parietal peritoneum, after having overlaid the lower third of the right kidney with its proximal ureter and gonadal vessels, the subpapillary lower half of the descending portion (second part) of the duodenum, and the whole horizontal portion (third part) and the lower third of the head of the pancreas reflect from back to front to form the inferior sheet of the transverse mesocolon; to the right, it adheres to the overlying fixed portion of the ascending mesocolon (both forming the right Toldt’s fascia); to the left, it continues with the right sheet of the mesentery root; at the bottom, it descends on the anterior aspect of the right psoas major muscle (Figure 1).

In keeping with this, Fredet’s fascia corresponds with the fusion plane between the inferior layer of the right transverse mesocolon and the anterior duodenopancreatic peritoneum.

More specifically, its anatomical boundaries are as follows: upward, the right root of the transverse mesocolon; downward, the inferior border of the duodenal horizontal portion (third part), below which Fredet’s fascia continues as the right of Toldt’s fascia; to the right, the medial margin of the hepatic flexure where Fredet’s fascia merges into the right fascia of Toldt’s; to the left, the right sheet of the mesentery root and immediately after, the Henle’s trunk and the medial border of the SMV.

Henle’s trunk is formed (in around 38.6% of all cases—Type I) by the convergence of the stomach-draining right gastroepiploic vein (RGEV), the anterior superior pancreaticoduodenal vein (ASPDV), and the colon-draining superior right colic vein (RCV) and drains into the superior mesenteric vein (SMV) at the inferior border of the pancreas head [29] (Figure 1). It is noteworthy that knowledge of the vascular anatomy and its variations is essential for CVL during open and minimally invasive CME.

Therefore, knowledge of Fredet’s fascia’s vascular surgical anatomical relationships is essential for colorectal surgeons to avoid accidental injuries to the superior mesenteric pedicle during right hemicolectomy with CME. Such knowledge then becomes indispensable in transferring CME from an open to a minimally invasive setting. It is also worth noting that laparoscopic and robot-assisted CME is an increasingly commonly performed procedure for the surgical treatment of colorectal cancer techniques by the authors of our systematic review, as shown in Table 6.

Moreover, Fredet’s fascia cannot be identified as Toldt’s fascia, which represents a distinct fascial plane apposed between the retroperitoneum and the right and left mesocola. However, it is also useful to note that the right Toldt’s fascia at the right inframesocolic margin of the second portion of the duodenum divides to merge into the *retropancreatic fusion* fascia or *fascia of Treitz*, dorsally, and the *sub-mesocolic pre-duodenal-pancreatic* fascia or *Fredt’s fascia*, ventrally.

This description fits well with classic anatomy textbooks such as Testut and Latarjet [55]: the fate of the primitive mesoduodenum (or dorsal duodenal mesentery) and of the ascending mesocolon, is the adhesion with the parietal peritoneum to form the *fascia of Treitz* (behind of duodenum) and the *right fascia of Toldt,* respectively. Conversely, in front of the subpapillary second duodenal portion and the lower third of the head of the pancreas, the fusion of the right inferior layer of the transverse mesocolon under its root, to the pre-duodenopancreatic peritoneum, constitutes the *sub-mesocolic pre-duodenal-pancreatic fascia or Fredet’s fascia* (Figure 1).

Regarding Fredet’s fascia’s anatomical terminology, it is correct to use the term “fascia” and not the term “area”. According to the *Terminologia Anatomica (TA) of the Federative Committee on Anatomical Terminology* [56], in fact, an *area* of the peritoneum corresponds to any part of the mesothelial surface of a single peritoneal *layer* (or *sheet*) while a *fascia* is a “generic” anatomical term that refers to a variety of the body’s soft fibrous connective tissue parts.

In particular, as mentioned above, Fredet’s fascia, as well as Toldt’s and Treitz’s fascia, are *fusion fasciae*, which only occur in the peritoneum during the rotation and posterior attachment of the primitive gut tube. Even more specifically, fusion fasciae are formed through the adherence between the mesothelium of the embryonic mesentery with the mesothelium of the embryonic retroperitoneum.

The concept of “fusion fascia” is indispensable when considering the relationship between the peritoneum and the mesenteries at the end of intestinal rotation around the axis of the superior mesenteric artery (SMA). Classic anatomical teaching holds that the intimate apposition of two mesothelially covered surfaces is followed by the disappearance of the mesothelial cell layer, leaving only the immature connective tissue of the developing peritoneum, designated as a “fusion fascia”.

In actual fact, as elegantly demonstrated by Culligan et al. [6], the two mesothelial cell layers persist on the deep surfaces of mesocolon (ascending and descending) and on the overlying retroperitoneum, and the connective tissue plane sandwiched between them represents the fusion fascia of Toldt (right and left, respectively).

In keeping with this, a *fusion fascia* is a thin connective tissue plane sandwiched between two mesothelial cell layers, which form a bloodless cleavage plane allowing the retroperitoneal portions of the bowel to be safely mobilized and also limits the spread of diseases.

Likewise, Fredet’s fusion fascia is a connective tissue plane situated between the deep mesothelial layer of the right transverse mesocolon and the mesothelium of the visceral duodenal-pancreatic peritoneum.

The present study suggests that the eponymous fascia of “Fredet” is poorly known and inconstantly used. Moreover, even if the debate over the use of eponyms in medicine continues, the anatomical terminology trend is to consider them obsolete. Their critics, in fact, contend that eponyms are inherently inaccurate and variable, frequently overlap in meaning, sometimes honor the wrong people, lack scientific descriptiveness, and are inconstantly interpreted in different countries.

In conclusion, they may be an impediment to efficient communication and learning.

Moreover, the synonyms utilized in the majority of studies such as “anterior pancreatic fascia” and “pre-duodenal fascia” or simply “duodenal fascia” are not consistently used across the literature and in any case refer to a partial (duodenum/pancreas) anatomical structure and not to its inframesocolic portion specifically.

Therefore, in order to avoid confusion and clarify this fascia for future use, we propose using a more comprehensive non-eponymous term, such as “sub-mesocolic pre-duodenopancreatic fascia”, which is centrally based on the anatomical structure in accordance with their embryonic development.

## 5. Conclusions

Our systematic literature review confirms that the term “Fredet fascia” is poorly or incorrectly used by some authors, likely due to the lack of both a comprehensive understanding of its anatomy and the standardization of its terminology.

As we are aware that the standardization of terminology is very important for international communication, research, and medical education, we suggest using a “descriptive” term such as *“sub-mesocolic pre-duodenopancreatic fascia*” instead. This term complies with the descriptive, topographical, informative, didactic, and communicative criteria of the International Anatomical Terminology, which, since 1933 and with few exceptions, has considered eponyms unofficial terms [56].

The authors’ explicit intention in recommending this term has been to facilitate clear and unambiguous international and interdisciplinary communication on this embryologic fusion fascia, in order to help standardize surgical practice, especially for more recent minimally invasive techniques, such as robotic and laparoscopic-assisted right-sided colon cancer surgery.

## Figures and Tables

**Table 1:** Types of article published.

Author, Year of Publication	Observational Study	Chapter of Book	Narrative Review	Case Report	Technical Note
Lin 2022 [54]	X				
Wedel 2022 [50]			X		
Tebala 2021 [52]					X
Peltrini 2021 [51]				X	
Matsuda 2020 [53]			X		
Garcia-Granero 2019 [9]	X				
Primo Romaguera 2019 [13]					X
Spinoglio 2018 [12]	X				
Siani 2017 [14]	X				
Siani 2017 [15]			X		
Spinoglio 2016 [16]	X				
Formisano 2016 [17]	X				
Cutini 2016 [18]		X			
Siani 2016 [19]			X		
Aseni 2016 [20]		X			
Siani 2015 [21]	X				
Galindo 2015 [22]		X			
Leroy 2014 [23]		X			
Trastulli 2013 [24]	X				
Yoo 2019 [25]		X			
Topgül 2013 [26]				X	
Lin 2013 [27]	X				
Ruotolo 2012 [28]			X		
Vasiliadis 2012 [29]			X		
Roscio 2012 [31]	X				
D’Annibale 2012 [32]	X				
Pugliese 2012 [33]		X			
Vasiliadis 2012 [30]			X		
Shuang 2011 [34]	X				
Milivoje 2010 [35]			X		
Benzoni 2010 [36]				X	
D’Annibale 2010 [37]	X				
Ocampo 2009 [38]			X		
Casadei 2009 [39]		X			
Huscher 2009 [40]		X			
Valle 2009 [41]					X
Huscher 2008 [42]		X			
Pugliese 2008 [43]	X				
Pugliese 2007 [44]	X				
Talamini 2006 [45]		X			
Madovanu 2005 [46]			X		
Natale 2005 [47]	X				
Ruotolo 2002 [48]		X			
Siani [49]		X			
Total	17	12	10	3	3

**Table 2:** Terms and synonyms of Fredet’s fascia used by the authors.

Author, Year of Publication	Fredet’s Fascia	Fredet Pre-Duodenal Pancreatic Fascia	Fredet’s Area	Pre-Duodenal Pancreatic Sub-Mesocolic Fredet Fascia	Fredet’s Right Transverse Mesocolon Fascia	Duodeno-Pancreatic Sub-Mesocolic Fascia
Lin 2022 [54]	X					
Wedel 2022 [50]	X	X	X			
Tebala 2021 [52]	X					
Peltrini 2021 [51]	X					
Matsuda 2020 [53]	X					
Garcia-Granero 2019 [9]	X					
Primo Romaguera 2019 [13]	X					
Spinoglio 2018 [12]	X					
Siani 2017 [14]		X				
Siani 2017 [15]	X					
Spinoglio 2016 [16]	X					
Formisano 2016 [17]	X					
Cutini 2016 [18]						
Siani 2016 [19]		X				
Aseni 2016 [20]		X	X			
Siani 2015 [21]		X				
Galindo 2015 [22]	X					
Leroy 2014 [23]	X					
Trastulli 2013 [24]	X					
Yoo 2019 [25]	X					
Topgül 2013 [26]	X					
Lin 2013 [27]			X			
Ruotolo 2012 [28]	X		X	X		
Vasiliadis 2012 [29]		X				
Roscio 2012 [31]	X					
D’Annibale 2012 [32]	X					
Pugliese 2012 [33]			X			

**Table 3:** Most common definitions reported of Fredet’s fascia.

Definition Reported of Fredet’s Fascia	Below the Root of the Mesocolon, the Fusion of the Inferior Lining of the Right Transverse Mesocolon to the Ventral Surface of the Pancreas (Pre-Duodenal Pancreatic Peritoneum)	Below the Root of the Mesocolon, the Fusion of the Inferior Lining of the Transverse Mesocolon (Right and Left) to the Ventral Surface of the Pancreas (Body and Tail)
Author, year of publication	Lin 2022 [54] Wedel 2022 [50] Tebala 2021 [52] Peltrini 2021 [51] Matsuda 2020 [53] Garcia-Granero 2019 [9] Primo Romaguera 2019 [13] Ruotolo 2012 [28] Ocampo 2009 [37] Huscher 2008 [42] Natale 2005 [47] Ruotolo 2002 [48]	Leroy 2014 [23] Yoo 2019 [25] Topgül 2013 [26]

**Table 4:** Surgical dissection maneuvers related to Fredet’s area or fascia.

Author, Year of Publication	Indication of Surgical Procedures	Fredet’s Area or Fascia
Lin 2022 [54]	Right colectomy	*“The surgeon first dissected the fusion fascia in the innermost area adjacent to the gastric antrum, entered the dorsal side of the fusion fascia of Fredet, and then gently expanded the surgical plane between the fusion fascia of Fredet and the visceral duodenal-pancreatic peritoneum in a medial-to-lateral direction”*
Wedel 2022 [50]	Right colectomy	*“The mesocolic fascia was attached to the parietal peritoneal fascia (‘fascia of Toldt’) along the parieto–mesocolic interface, and further cranially to the pre-duodenopancreatic fascia along the mesocolic–duodenopancreatic interface (‘space of Fredet’)”*
Tebala 2021 [52]	Right colectomy	*“After the Duodenal Window is opened, Fredet’s and Toldt’s fasciae are dissected, usually in a bloodless fashion, and can be extended up to the lateral Toldt’s line, to the hepatic flexure and to the gastroepiploic vessels, thus allowing complete preparation of the right mesocolon”.*
Garcia-Granero 2019 [9]	Right colectomy	*“Fusion fascia of Fredet: adhesion plane between visceral peritoneum of the mesocolon of ascending colon and hepatic colonic flexure and the visceral peritoneum of duodenum and pancreas”*
Primo Romaguera 2019 [13]	Duodenopancreatectomy	*“La fascia de Fredet, coalescencia entre el mesocolon ascendente y el peritoneo visceral duodeno-pancreático”*
Cutini 2016 [18]	Gastrectomy	*“During this maneuver the assistant’s grasper pulls up the great curvature of the antropyloric region while the surgeon, dissecting Fredet’s area, exposes the superior mesenteric vein”*
Aseni 2016 [20]	Transplantation	*“The correct line of dissection continues among Gerota’s fascia, Toldt’s fascia, and the preduodenopancreatic fascia or Fredet’s area”*
Lin 2013 [27]	Gastrectomy	“*The dissection moved to the hepatic flexure and the pylorus. The right gastroepiploicvein was divided between titanium clips flush with theHenle’s trunk and ended up in the Fredet area, wheregroup 14v was removed”*
Ruotolo 2012 [28]	Surgical anatomy	“*Below the root of the mesocolon, the fusion of the primary preduodenopancreatic peritoneum (anterior mesoduodenum) with the lower leaflet of the right transverse mesocolon generates the submesocolicpreduodenopancreatic fascia or Fredet’s area”*
Pugliese 2012, 2007 [33,44]	Right colectomy	*“The right gastroepiploic artery is sectioned at its origin from the gastroduodenal artery, just above the pancreatic head. This allows clearance of station 6 and of Fredet’s area, where lymph node station 14v is removed”*
Shuang 2011 [34]	Right colectomy	*“The right gastroepiploic vein was divided between titanium clips flush with the Henle’s trunk and ended up in the Fredet area, where group 14v was removed”.*

**Table 5:** Surgical anatomy of Fredet’s fascia.

Surgical Step	Surgical Relevance of Fredet’s Fascia	Author
Robotic/Laparoscopic Right Colectomy		
*“A complete mesocolic excision is performed by dissecting along the plane between the intact dorsal mesocolon of the hepatic flexure and the Fredet preduodenopancreatic fascia”*	*“The dissection is sharp in this area* *This plane is bloodless (completely avascular)”*	Lin 2022 [54] Wedel 2022 [50] Tebala 2021 [52] Garcia-Granero 2019 [9] Primo Romaguera 2019 [13] Spinoglio 2018, 2016 [12,16] Formisano 2016 [17] Siani (2015–2017) [14,19,21] Huscher 2009 [40]
Duodenopancreatectomy		
*“Dissection along the plane of the area of Fredet allows the exposure of the anterior aspect of the head of pancreas dorsal to the mesentery root, the second portion of duodenum below the papilla of Vater and the third and fourth portions of duodenum* *This maneuver is also one of the main steps of the Cattel-Braash maneuver”*	*“Dissection of the fascia of Fredet is certainly more challenging if compared with dissection along the right side of Toldt and the fascia of Trietz”*	Galindo 2015 [22] Talamini 2006 [45]
*“Fredet’s fascia is left intact during the dissection”*	*“Enbloc”resection of the pancreatic head covered anteriorly by Fredet’s fascia and posteriorly by Treitz’s fascia*	Huscher 2009 [40]
Laparoscopic gastrectomy		
*“The gastrocolic ligament is divided along the border of the transverse colon and the dissection moved to the hepatic flexure and the pylorus at area of Fredet”*	*“The dissection of Fredet’s area exposes the superior mesenteric vein. The dissection proceeds in order to identify the right colic vein, the gastroepiploic vein, and Henle’s trunk. In this manner the nodes of 14v station are removed, and the right gastroepiploic vein is clipped and sectioned”*	Cutini 2016 [18] Shuang 2011 [34]

**Table 6:** Landmarks of vascular surgical anatomy in the Fredet’ fascia.

Author, Year of Publication	Vascular Landmarks	Type of Surgery
Lin 2022 [54]	*“Using the projection of the superior right colic vein (SRCV) on the fusion fascia of Fredet as a landmark, the surgical plane was expanded medially to expose the gastrocolic trunk of Henle (GCTH), and the nonvascularized mesocolic area was expanded on the left side of the root of the middle colonic vessels, completing the dissection of the surgical area of the GCTH* [14,15] *(SAGCTH), defined as the area of the superior mesenteric vein (SMV) located at the head of the pancreas and including the venous confluence of the right gastroepiploic vein (RGEV), anterosuperior pancreaticduodenal vein (ASPV), and SRCV”*	*Laparoscopic right colectomy*
Tebala 2021 [52]	*“Fredet’s fascia is an important landmark during anatomical mesocolic dissection of the right and proximal transverse colon and its preparation is one of the most challenging steps of this manoeuvre, mostly due to the risk of bleeding from the small tributaries to the superior mesenteric vein, including Henle’s trunk”*	*Laparoscopic right colectomy*
Matsuda 2020 [53]	*“Fusion fascia of Fredet as an essential embryological landmark during LRH, which corresponds to the plane between the ascending mesocolon and the visceral duodenal-pancreatic peritoneum”*	*Laparoscopic right colectomy*
Garcia-Granero 2019 [9]	*“We found that the medial limit of the fascia of Fredet is represented by the SMV and GCTH. In fact, the dissection of the fascia of Fredet allowed us to enter this area safer, with better exposure, and it offered a correct mesocolic plane, thus reducing the risk of injuries and bleeding from the SMV. The optimal knowledge of this anatomical landmark is crucial to avoid injuries to the SMV, which can lead to life-threatening complications”*	*Laparoscopic right colectomy*
Primo Romaguera 2019 [13]	*“The anatomical knowledge of Fredet’s fascia is necessary to achieve D3-lymphadenectomy in right colon cancer and can reduce the risk of postoperative complications”*	*Laparoscopic right colectomy*
Cutini 2016 [18]	*“The same manner the surgeon completes coloepiploic detachment moving the dissection toward the pylorus and achieving the lymphadenectomy of station 4a. During this maneuver the assistant’s grasper pulls up the great curvature of the antropyloric region while the surgeon, dissecting Fredet’s area, exposes the superior mesenteric vein. The dissection proceeds in order to identify* *the right colic vein, the gastroepiploic vein, and Henle’s trunk. In this manner the nodes of 14v station are removed, and the right gastroepiploic vein is clipped and sectioned”*	*Laparoscopic gastrectomy*
Lin 2013 [27]	*“The right gastroepiploic vein was divided between titanium clips flush with the Henle’s trunk and ended up in the Fredet area, where group 14v was removed”*	*Laparoscopic gastrectomy*
Pugliese 2012, 2007 [33,44] Shuang 2011 [34]	*“The right gastroepiploic artery is sectioned at its origin from the gastroduodenal artery, just above the pancreatic head. This allows clearance of station 6 and of Fredet’s area, where lymph node station 14v is removed”*	*Laparoscopic gastrectomy*
Huscher 2008 [42]	*“The only opening in Fredet’s fascia is right before the superior mesenteric vein (SMV), at the level where the Henle (grastro omental) trunk merges into the vein”*	*Duodenopancreatectomy*
Moldovanu 2005 [46]	*“The head of the pancreas has relations with the gastroduodenal arteries, the superior pancreaticoduodenal artery, the right gastroepiploic, the superior right colic and with the Fredet fascia”*	*Duodenopancreatectomy*

## Data Availability

The literature was searched using PubMed, Scopus, and Web of Science. The data presented in this study are available on request.
